# The Devastating Economic Impact of COVID-19 on Older Black and Latinx Adults: Implications for Health and Well-Being

**DOI:** 10.1093/geroni/igab046.752

**Published:** 2021-12-17

**Authors:** Catherine Garcia

**Affiliations:** University of Nebraska - Lincoln, Lincoln, Nebraska, United States

## Abstract

The ongoing COVID-19 pandemic and subsequent economic recession have wreaked havoc on the United States’ economy and brought to the forefront stark racial and ethnic inequalities in our society. Older Black and Latinx adults are particularly hard hit by the pandemic as they have relatively lower levels of income and wealth to protect against crises. This study used data from the 2020 COVID-19 module of the Health and Retirement Study, to highlight how the COVID-19 pandemic has economically impacted older Black, U.S.-born Latinx and foreign-born Latinx adults. Results show the pandemic has economically devastated older Black and Latinx adults across a host of economic factors, with foreign-born Latinx experiencing greater economic hardships relative to other groups. Our findings document stark inequalities that are being exacerbated by the pandemic. We discuss the implications of the economic shocks of the pandemic for the health and well-being of older Black and Latinx adults.

